# Dr. Fitzpatrick on Scarlatina

**Published:** 1842-04-16

**Authors:** Thomas Fitzpatrick

**Affiliations:** Licentiate of the King and Queen’s College of Physicians


### ANALECTA.

*Observations on Scarlatina.* By THOMAS FITZPATRICK, M. D., Licentiate of the King and Queen's College of Physicians.—[Scarlatina is a disease of such frequent occurrence, that any practical hints tending to diminish in any degree its mortality, are looked to with interest. The author of these observations has certainly not been more fortunate than his predecessors in falling upon a plan of treatment which may be considered as the best adapted to the treatment of the disease. To certain conditions it is, however, applicable, and to precisely those which accompany the most fatal cases. The prophylactic treatment by mercury certainly promises as well as the belladonna; whether it is entitled to more confidence, time must show.]

"Let us take the following example: A child is attacked with fever and sore throat; scarlatina is apprehended; the child is purged on the first day; on the second, the feverish symptoms are not alleviated, and the throat is probably worse, the body being covered with the eruption. The child is leeched, purged again, and tartar emetic solution is then administered; the little patient is first in some degree exhausted by the application of leeches; during this state the action of the purgative commences; the child is carelessly taken out of bed, not to answer the call of nature, but of medicine, and is exposed during its operation to the cold air. The tartar emetic is now given, and nausea and vomiting are added to the ante-febrile agents. Still the fever is not subdued; the pulse continues rapid, but weak; the eruption begins to disappear, the child, from being restless, becomes remarkably quiet, and a train of formidable symptoms follows. Having witnessed such results, I have adopted a different mode of practice; I place the patient, if possible, in a large and airy room, directing, after the appearance of the eruption, that the surface of the body should be carefully at all times covered by the bed-clothes, regulating their quantity by the temperature of the atmosphere, but taking care that an equal temperature is always maintained, moderating the heat and uneasiness in the skin by frequent tepid sponging, and administering aperients only sufficient to keep up the regular action of the bowels; by this mode of proceeding (being of course prepared to meet inflammation which might arise) I feel assured, that severe forms of the disease terminated favourably, which, under a more decidedly antiphlogistic treatment, would have assumed dangerous appearances. Let me not understood as advocating the old heating and stimulant plan of treatment, nor deprecating depletion, when circumstances demand it; but such circumstances generally prove the exception, not the rule. In proof of this assertion, I may observe, that Dr. Williams, in his work on Morbid Poisons, mentions, that in 241 cases, treated at the London Foundling and Fever Hospitals by bleeding and active depletion, the deaths were one in six, while in 555 cases treated by aperients and mineral acids, the deaths were one in twenty-two. We have, however, unfortunately too well established evidence, that in some cases of this malady, all modes of treatment are unavailing; too many instances have occurred of whole families being carried off, notwithstanding the unceasing efforts of the most experienced practitioners. It is to such cases occurring in families that I wish to direct the attention of the society." * * *

"I was not aware of any remedy having been suggested under such circumstances, with the exception of belladonna, and in this I had no confidence, particularly as I considered it highly probable, that, from the necessary exposure to contagion, the children were, at the time, under the influence of the miasma. The extraordinary effect of rapid mercurial action, in modifying the character of the disease, as exhibited in the case of the lady already alluded to, influenced me in concluding, that the exhibition of mercury in daily alterative doses, prior to the establishment of the febrile stage, would place the children in the most favourable position for the production of the full effect of this remedy, *at a very early period of the disease*, should the character of the inflammatory symptoms require such a proceeding. From the high repute which the mineral acids possess in the treatment of scarlatina,

I also determined on giving muriatic acid, having, besides, in view its tonic effect. I considered, that by cautious observation of the effects of the mercury, and by taking care that it was not given to such an extent as to produce its usual marked effects on the system, no possible injury could accrue to the child if he escaped scarlatina; while if it became developed he would not be in a more unfavourable state for the resistance of its influence. On the contrary, it might reasonably be supposed, that even if the effect of the remedies was merely the promotion of the natural functions of the alimentary canal, such a result would be more beneficial than otherwise; but another important consequence might follow, namely, the production of an altered condition of the individual's constitution, which would modify the character of the disease, it being remarkable, that the most apparently strong natural constitution, so far from offering due resistance to scarlatina, serves rather as an indication of its probable severity. My observation leads to the conclusion, that children of a delicate and even scrofulous constitution, frequently present the disease in a milder form, and certainly the most extraordinary recoveries from the greatest danger that I witnessed, occurred in individuals of this class. Influenced by these considerations, I visited the children on the following day, (June 19th,) and ordered a mixture containing dilute muriatic acid, mucil. of gum arabic and water, the dose being so regulated, that the eldest child took five drops of the acid three times a day, and the younger one a proportionate dose. I also directed three grains of blue pill for the boy, and half that quantity for his sister to be taken at bed-hour, and advised that they should be allowed to take their usual exercise, and that their diet should be light and nutritious." * * * *

"The younger child was attacked on the morning of the 23d; the symptoms bore a resemblance to those of her brother, but in a milder degree; the treatment was consequently less energetic, leeches not being required. In neither case was it necessary to continue the calomel after the first twenty-four hours, nor was there any evidence of its having affected the system. In the consideration of the result of treatment in the foregoing cases, it may be questioned, whether they might not have proved as favourable without any preparatory treatment, it being occasionally observed, that variations in the character of the disease occur in its progress through families. While we are ignorant of the cause of such variations, it is quite idle to attempt the solution of this question. Although it is admitted, that they are occasionally observed, yet medical authorities of great eminence are opposed to their general probability. Dr. Willan states, 'that when scarlatina spread widely, it exhibited, in the different persons affected, every variety and gradation of appearance, from the slightest to the most malignant form of the disease, yet, during its diffusion through some large families and schools, he had seen it uniformly retain the series of symptoms which occurred in the first patient with nearly the same degree of fever.'" * * *

"I do not advocate the doctrine that mercury is a specific in scarlatina, for the simple reason, that I have witnessed cases in which ptyalism was established, eventually proving fatal, but it was at an advanced period of the disease, when the patients became mercurialized, and even then, a temporary improvement was produced, followed, however, by an aggravated fatal termination. I have before remarked, that one object I had in view in administering mercury in alterative doses previous to the febrile stage, was to place the patient in that position whereby, if the character of the inflammation demanded it, I could with greater probability produce a rapid mercurial effect, with the hope of *arresting the inflammatory action in its progress*, an object which, under ordinary circumstances, can scarcely be produced, if the febrile stage has existed for any lengthened period, because this state of the system bears an analogy to bad typhoid fever, in presenting the greatest difficulty of bringing the constitution under the influence of mercury ; indeed, the attempt to induce mercurial action in scarlatina after the febrile stage has been fully developed, and the local inflammation has arrived at a degree of intensity, is rather injurious than beneficial. I shall not stop here to inquire whether the first link in the chain of morbid action is connected with the great nervous centres, or the mucous membrane, but I feel satisfied, that the *early arrest* of inflammation in the mucous membrane is most desirable; and we have positive evidence that such an effect can be produced by creating a sudden new impression on the part, even though the irritated or inflammatory state may arise from a specific poison ; this difference being applicable to scarlatina, that mere local treatment fails, unless accompanied by remedies which produce an effect on the general constitution."—*Dublin Journal for March.*

